# Computational Study of Subdural Cortical Stimulation: Effects of Simulating Anisotropic Conductivity on Activation of Cortical Neurons

**DOI:** 10.1371/journal.pone.0128590

**Published:** 2015-06-09

**Authors:** Hyeon Seo, Donghyeon Kim, Sung Chan Jun

**Affiliations:** School of Information and Communications, Gwangju Institute of Science and Technology, Gwangju, South Korea; University of Houston, UNITED STATES

## Abstract

Subdural cortical stimulation (SuCS) is an appealing method in the treatment of neurological disorders, and computational modeling studies of SuCS have been applied to determine the optimal design for electrotherapy. To achieve a better understanding of computational modeling on the stimulation effects of SuCS, the influence of anisotropic white matter conductivity on the activation of cortical neurons was investigated in a realistic head model. In this paper, we constructed pyramidal neuronal models (layers 3 and 5) that showed primary excitation of the corticospinal tract, and an anatomically realistic head model reflecting complex brain geometry. The anisotropic information was acquired from diffusion tensor magnetic resonance imaging (DT-MRI) and then applied to the white matter at various ratios of anisotropic conductivity. First, we compared the isotropic and anisotropic models; compared to the isotropic model, the anisotropic model showed that neurons were activated in the deeper bank during cathodal stimulation and in the wider crown during anodal stimulation. Second, several popular anisotropic principles were adapted to investigate the effects of variations in anisotropic information. We observed that excitation thresholds varied with anisotropic principles, especially with anodal stimulation. Overall, incorporating anisotropic conductivity into the anatomically realistic head model is critical for accurate estimation of neuronal responses; however, caution should be used in the selection of anisotropic information.

## Introduction

Electrical cortical stimulation (CS) is an intriguing electrotherapy designed to expedite neuronal modulation in the brain cortex through the regulated input of current. It has been applied as a treatment for chronic pain [1–4], rehabilitation [5–8], Parkinson’s disease [2,9–11], essential tremor [2], and other brain disorders [12,13]. CS can be categorized into invasive and noninvasive approaches depending on whether or not the input devices are implanted. It has been reported that invasive approaches provide performance superior to noninvasive methods in such disabilities as chronic pain and movement disorders [12]. Whether input electrodes are implanted epidurally (on the dura mater) or subdurally (under the dura mater), CS is called epidural cortical stimulation (ECS) or subdural cortical stimulation (SuCS). In particular, SuCS has two advantages, in that it is easy to target the desired brain region and allows neuronal activation to be evoked with a relatively less intense current than in ECS. Furthermore, SuCS is less invasive than deep brain stimulation (DBS) and can be an alternative to ECS for some patients who suffer from advanced cortical atrophy due to duro-cortical separation.

Until now, CS has been employed with less tuned stimulation parameters, which are determined primarily by clinical experience due to the lack of complete understanding about how the current input propagates in the cortex. Furthermore, the large number of potential combinations of stimulation parameters leads to uncertain therapeutic outcomes [13]. Recent research has been conducted to analyze the underlying mechanism of CS using computational approaches to determine optimal stimulation parameters. Such studies have led to improvement in therapeutic effects by inferring neuronal excitability from the estimated electric field (EF) or current density (CD) induced by input current [14–32]. To elucidate the spatial extent of induced EF/CD, a volume conduction model of the human head is needed, such as a simplified or realistic head model. In recent years, a realistic head model has been used widely to improve the spatial accuracy of stimulation, because magnetic resonance (MR) images can be used to reflect brain anatomy [17–32]. Furthermore, these studies have incorporated the anisotropic conductivity derived from diffusion tensor (DT) imaging into their head models [21–27] and have shown variation in the spatial patterns and strength of induced EF/CD, as well as alteration of current flow in directions parallel to the white matter (WM) fiber tract according to the inclusion of anisotropy. However, most investigations using anisotropic conductivity with realistic head models have focused on noninvasive approaches, while invasive studies are rare. The most recent study examined the effects of SuCS on anisotropic conductivity using a realistic head model [33], but this investigation using a realistic head model was limited to macroscopic or mesoscopic estimations of EF/CD in the cortex, rather than microscopic estimations at the neuronal level.

The simple quantification of EF or CD is insufficient to explain complex neuronal modulation, because responses of cortical neurons vary depending on their shape, size, location, and orientation [34–36]. For a detailed investigation at the neuronal level, compartmental cortical neuronal models coupled with the head model are required. However, due to the complex brain geometry of the realistic head model, those neuronal models are usually combined with a simplified head model (extruded slab model). The simplified extruded slab model represents the typical precentral gyrus region and this simple geometry makes it easy to couple neuronal and brain models in a straightforward manner. However, due to possible modeling error (anatomical mismatch of the volume conduction model of simplified and real head models), this method is expected to produce non-negligible discrepancies between computational and empirical (ground truth) electric fields, which can be a crucial factor in determining whether or not neuronal activation takes place [17,22,37]. In a recent study, the effects of transcranial direct current stimulation (tDCS) on cortical neurons were assessed using a realistic head model [35], in which neuronal activation was investigated under the assumption that tDCS produces uniform electric fields in gray matter (GM); thus, complex brain geometry effects have not been considered directly in neuronal models.

To the best of our knowledge, the computational study of SuCS using an anatomically realistic head model has not been investigated at the neuronal level. In response to this need, the significance of neuronal activation by a realistic electric field produced by SuCS was reported by our group at the EMBC 2013 [38]; in preliminary work, small numbers of neurons in the computational domains were considered concisely for comparison between the simplified extruded slab model and realistic head model. However, this study did not consider anisotropic conductivity in the WM properly and the number of neurons was too small to represent the typical neurons in the brain cortex. For these reasons, in this work, we constructed an anatomically realistic full head model with anisotropic conductivity acquired from DTI and a large number of neuronal models. Two types of layer 3 and layer 5 pyramidal neuronal models were developed for uniform distribution around the precentral gyrus.

Our goal was to investigate the effect of anisotropic conductivity on neuronal activation produced by SuCS using the anatomically realistic head model with three types of stimulation polarities (cathodal, anodal, and bipolar). Their influence was explored by quantitative estimation of excitation thresholds that evoke neuron activation, as well as the percentage of neurons excited and the minimum threshold.

## Methods

### Construction of the anatomically realistic head model

**Fig 1:**
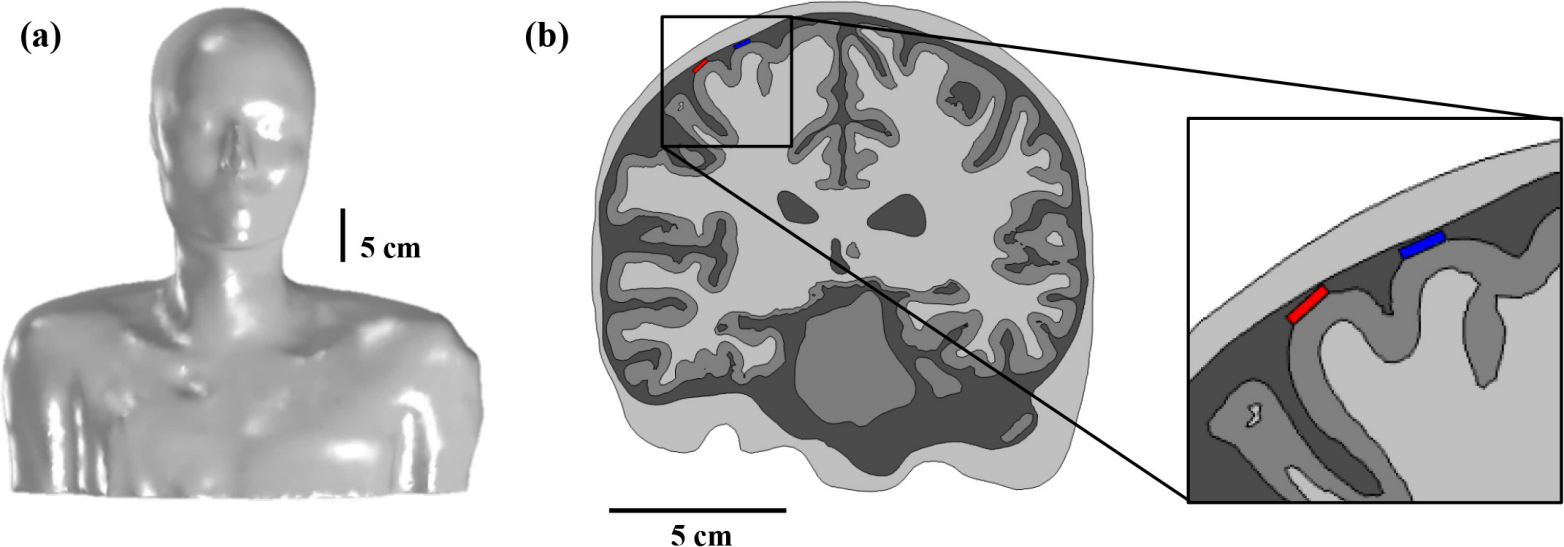
The shape of the anatomically realistic head model. (a) Whole head model and (b) cross-section of head model for subdural cortical stimulation are shown. Red and blue dots on the cortex represent implanted electrodes.

A volume conduction model of the human head, including stimulus electrodes, was developed using a brain and whole human body MRI acquired from SimNIBS [22] and the visible human project of Korea [39] (Fig 1). We note that these human MRI data are anonymized, de-identified, and are publicly accessible. For this reason, the institutional review board (IRB) approval of Gwangju Institute of Science and Technology (GIST) was not required for this study. We segmented WM, GM and the cerebellum based on FreeSurfer [40,41], and used FMRIB FSL [42] to extract CSF, ventricles, skull and scalp. The shape of the upper body was extracted using Seg3D [43]. Before creating tetrahedral finite element meshes from surface meshes, we attached two electrodes to the precentral gyrus representing the hand area [44]. We designed two disc-typed electrodes (height = 0.1 mm; diameter = 4 mm) to be covered with substrates (height = 1.1 mm; diameter = 5 mm), and these two electrodes were implanted subdurally 13 mm apart. The substrate was constructed by considering the clinical use of strip-type electrodes, but we limited the substrate to surround a disc-type electrode because of the difficulty of modeling strip-type electrodes in the brain [19]. Then, we considered upper electrode (blue in Fig 1(B)) only as an active electrode for monopolar stimulation (anodal or cathodal); upper electrode as a cathode and bottom electrode (red in Fig 1(B)) as an anode were considered for bipolar stimulation. In a clinical situation, a pulse generator is implanted in the pectoral region; thus, a disc-type reference electrode (height = 12 mm; diameter = 11.5 mm) was modeled on the chest. Finally, we generated optimized volumetric mesh using iso2mesh [45] and tetgen [46].

These 3D computational models were input in COMSOL Multiphysics (v4.3b, COMSOL, Inc., Burlington, MA, USA) and solved numerically by the finite element method. The number of total tetrahedral mesh elements was 8.8 million. Smaller elements were used primarily around the cortex near electrodes and then they became larger toward the chest area. The bi-conjugate gradient method (a relative tolerance of 1 × 10^−6^) with preconditioning of an algebraic multigrid was used as a solver.

### Conductivity assignment

Anisotropic conductivity assigned to the WM was constructed in the anatomically realistic head model. Except for WM, all other tissue types were modeled as isotropic. The conductivities for each layer, which were obtained from the literature [15,18,47,48], are tabulated in Table 1.

**Table 1:** Conductivities of tissues and electrodes [15,18,47,48].

Compartment	Conductivity (S/m)
Substrate conductivity	0.1 × 10^−9^
Electrode conductivity	9.4 × 10^6^
Scalp	0.465
Skull	0.01
Dura mater	0.065
CSF	1.65
Gray matter	0.276
White matter (isotropic)	0.126
White matter (parellel to fibers)	1.1
White matter (perpendicular to fibers)	0.13

In the anatomically realistic head model, it was difficult to determine the major anisotropic direction of WM, because fibers were distributed in a complex manner due to the complexity of brain geometry. Accordingly, we derived an electrical conductivity tensor of WM for the anatomically realistic head model from the water diffusion tensor, as measured by diffusion tensor magnetic resonance imaging (DT-MRI), assuming that conductivity and diffusion tensors share the same eigenvectors [49]. In this case, we shared only eigenvectors and then set the eigenvalue along the largest and perpendicular eigenvectors in three ways. First we used artificial anisotropy with a fixed value (fixed value anisotropy). We set the eigenvalue along the largest eigenvector (σ^long^, longitudinal direction) as 1.1 S/m, and the perpendicular eigenvectors (σ^trans^, transverse direction) as 0.13 S/m, as were shown in Table 1. Next, we modeled the conductivity tensor σ of the WM as follows:

$$\sigma =\text{S diag}\left(\sigma^{\text{long}},\sigma^{\text{trans}},\sigma^{\text{trans}}\right)\;{\text{S}}^{\text{T}}$$ (1)

where S is the orthogonal matrix consisting of unit length eigenvectors of the diffusion tensor.

In addition to obtaining a more generalized conductivity tensor, we used a direct transformation approach with volume normalization (volume normalization anisotropy) and artificial anisotropy with volume constraint (volume constraint anisotropy) [50,51].

The volume normalization anisotropy maintains the mean conductivity of the tensors at the isotropic WM value (σ^iso^). It calculates normalized eigenvalues $\sigma_{{\text{v}}_{\text{i}}}^{\prime }$ as

$$\sigma_{{\text{v}}_{\text{i}}}^{\prime }=\sigma_{{\text{v}}_{\text{i}}}\frac{\sigma^{\text{iso}}}{\sqrt[{3}]{\sigma_{{\text{v}}_{1}}\sigma_{{\text{v}}_{2}}\sigma_{{\text{v}}_{3}}}}$$ (2)

where $\sigma_{{\text{v}}_{\text{i}}}$ is the conductivity tensor eigenvalue of the DTI data.

The volume constraint anisotropy fixed the artificial anisotropy ratio to the eigenvalues by calculating

$$\sigma^{\text{long}}=\sqrt[{3}]{{\left(\sigma^{\text{iso}}\right)}^{3}{\text{r}}^{2}}$$ (3)

$$\sigma^{\text{trans}}=\sqrt[{3}]{{\left(\sigma^{\text{iso}}\right)}^{3}/\text{r}}$$ (4)

where r is the anisotropic factor (r = 2 if 2:1, r = 5 if 5:1, and so on). We varied the anisotropic factor from 2, 5, and 10 to 100; the conductivity values are tabulated in Table 2.

**Table 2:** Longitudinal and transverse conductivity of WM tensor elements calculated using the artificial anisotropy with volume constraint according to anisotropic factors (r).

	r = 2	r = 5	r = 10	r = 100
**σ** ^***long***^	0.200	0.368	0.585	2.714
**σ** ^**trans**^	0.100	0.074	0.059	0.027

### Compartmental models of pyramidal neurons

In this work, layer 5 (L5) and layer 3 (L3) pyramidal neuronal models were constructed. We used the detailed morphology and electrical properties from the cat visual cortex [52] and lengthened the neuronal models by 60% to fit human brain geometry [34]. Briefly, low density Na+ channels were present in the soma and dendrites, and high density channels were present in the axon hillock and initial segment. The axon and soma included fast K+ channels, while dendrites did not. Slow K+ channels and high-threshold Ca2+ channels were present in both soma and dendrites. Simulations were performed in the NEURON environment [53].

Due to the complexity and limitations of computational resources, such neurons could not be constructed explicitly in the 3D computational models. Thus, two kinds of L5 and L3 neuronal models were positioned virtually in the anatomically realistic head model. We computed the fields of stimulus-induced potentials distributed in the head model, and then the electric potentials were applied to each compartment of the neuronal models by extracellular stimulation. We applied a monophasic rectangular stimulating pulse 100 μs in duration, and defined the excitation threshold of a neuron when the membrane potential at one of the nodes in the corresponding neuronal model was raised by 70 mV or more above the resting potential [54].

**Fig 2:**
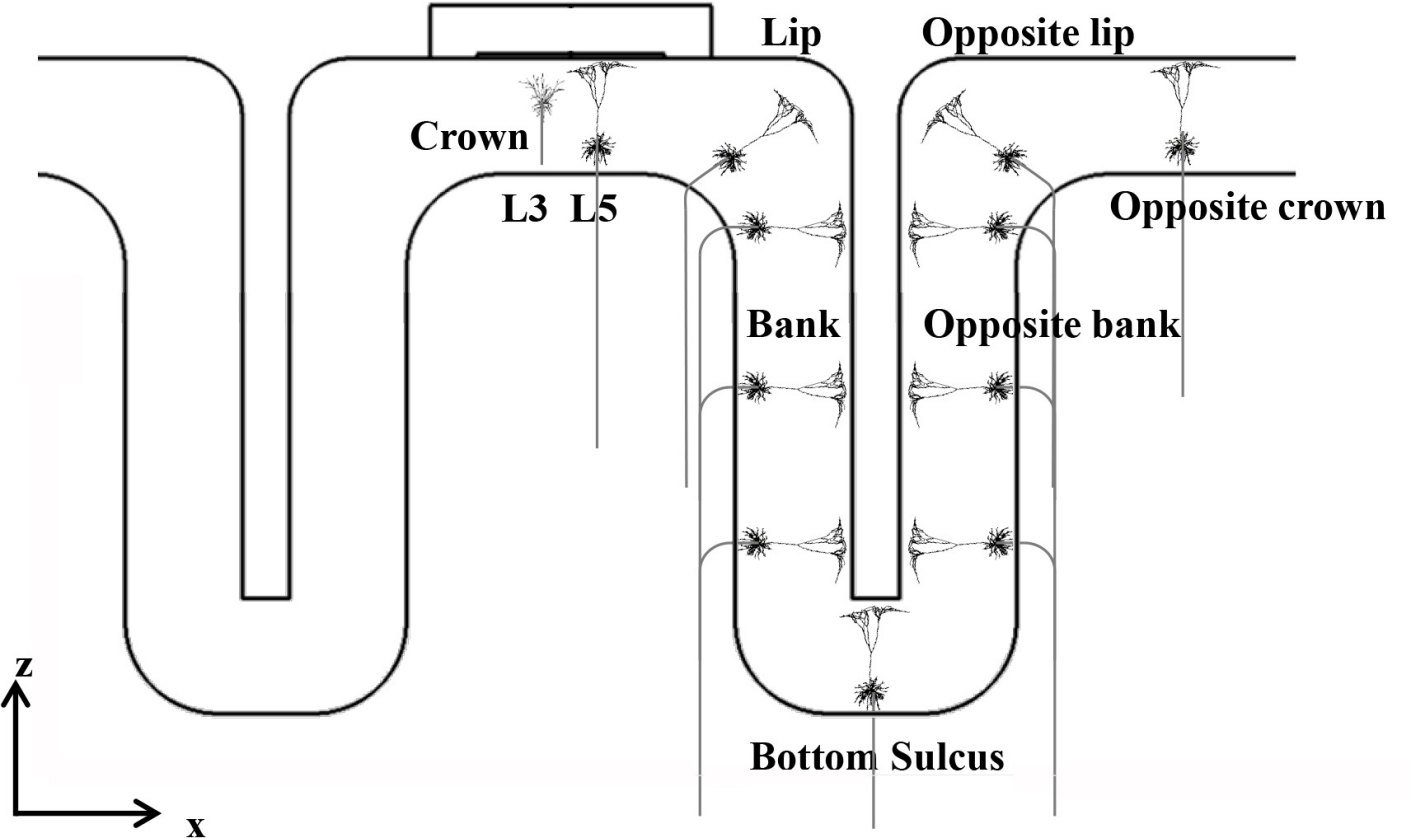
Schematic view of the neuronal model morphology and placement. Orientations of layer 3(L3) and layer 5(L5) pyramidal neuronal models are shown, with bends in different locations. They represent only one aspect of the uniform neuronal models.

Rather than modeling the neuronal models within the whole brain area, we designated the region of interest (ROI) as a regular hexahedron within a volume of 5 × 10^3^ mm^3^ with the electrodes in the middle, and placed two types of L5 and L3 neuronal models uniformly in the ROI to reduce superfluous computations. The orientation of the neuronal model was perpendicular to the cortex. L5 neurons curved beyond the boundary between GM and WM, while L3 models were located within the cortex. The soma of L5 and L3 neuronal models were placed 0.6 mm and 1.8 mm above the boundary between the GM and WM [34]. These neuronal models are illustrated in detail in Fig 2.

**Fig 3:**
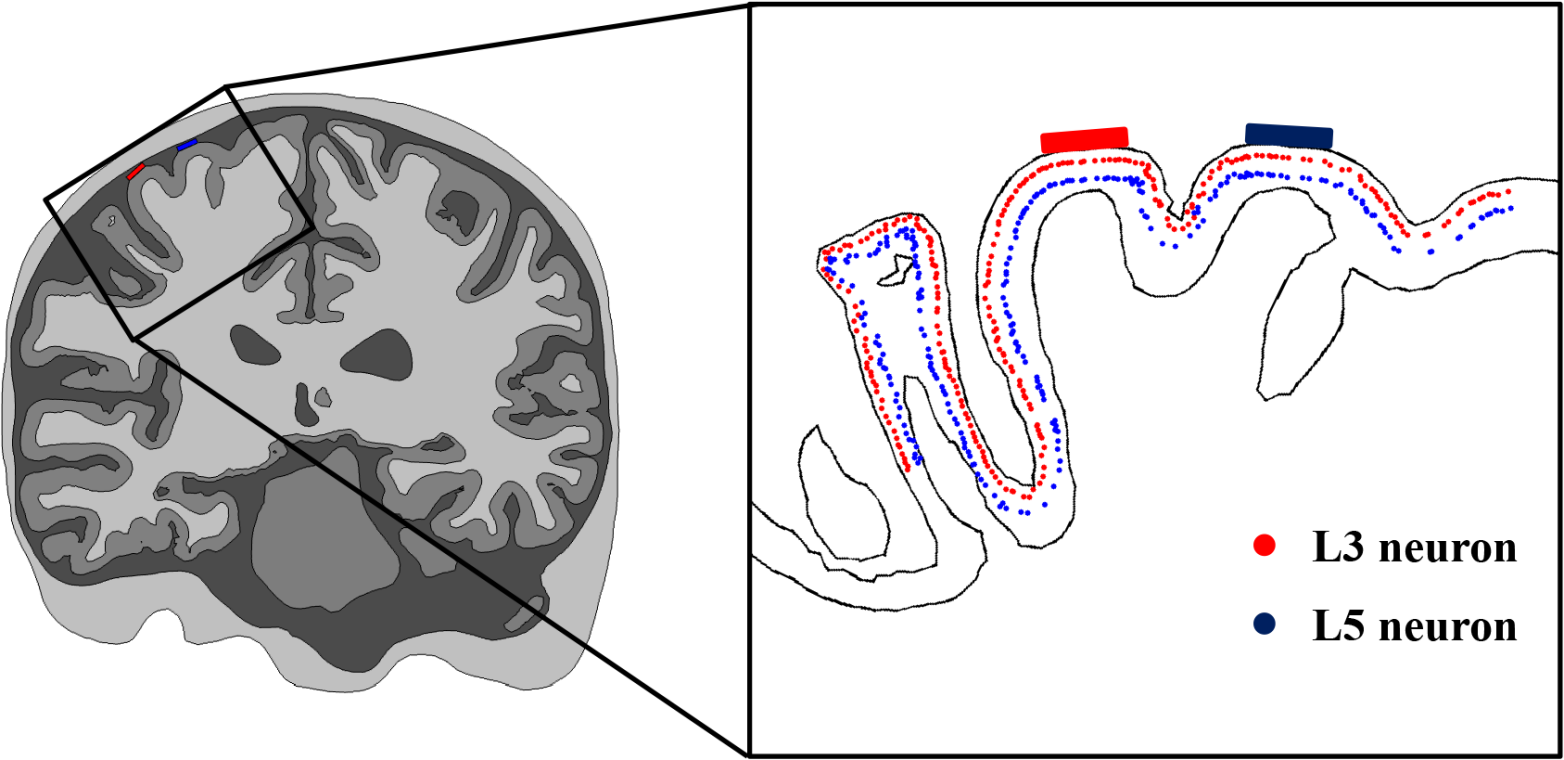
Placement of L5 and L3 pyramidal neuronal models in the anatomically realistic head model. Locations of L5 and L3 somata are marked as colored dots (blue: L5, red: L3) in cross-section passing through two electrodes.

Each of the L5 and L3 neuronal models was allocated in each triangular element comprising the GM surface and aligned with the normal direction of this element. Therefore, we constructed a total of 12,824 neuronal models each (L5 or L3) and distributed them uniformly, as shown in Fig 3. This process was implemented in MATLAB (MathWorks, Natick, MA, USA) and COMSOL 4.3b with MATLAB.

## Results

First, to investigate the influence of anisotropic conductivity on neuronal activation, we compared the effects of simulating the anisotropic and isotropic conductivity using the anatomically realistic head model. Next, to gain further insight into the anisotropic conductivity, three methods were incorporated to determine the anisotropic information. We analyzed the effect of anisotropic conductivity on pyramidal neuronal activation by increasing the stimulus amplitude up to 100 mA. Even though this amplitude is impractical, in the light of the advantages of the simulation study, we observed the tendency for neuronal responses up to this higher amplitude.

### Comparison between anisotropic and isotropic conductivities

We assessed the changes induced by tissue anisotropy relative to the equivalent isotropic model. For the isotropic model, WM conductivity was assigned to 0.126 S/m, while fixed value anisotropy (1.1 S/m and 0.13 S/m conductivity values in longitudinal and transverse directions, respectively) was applied to the anisotropic model.

**Fig 4:**
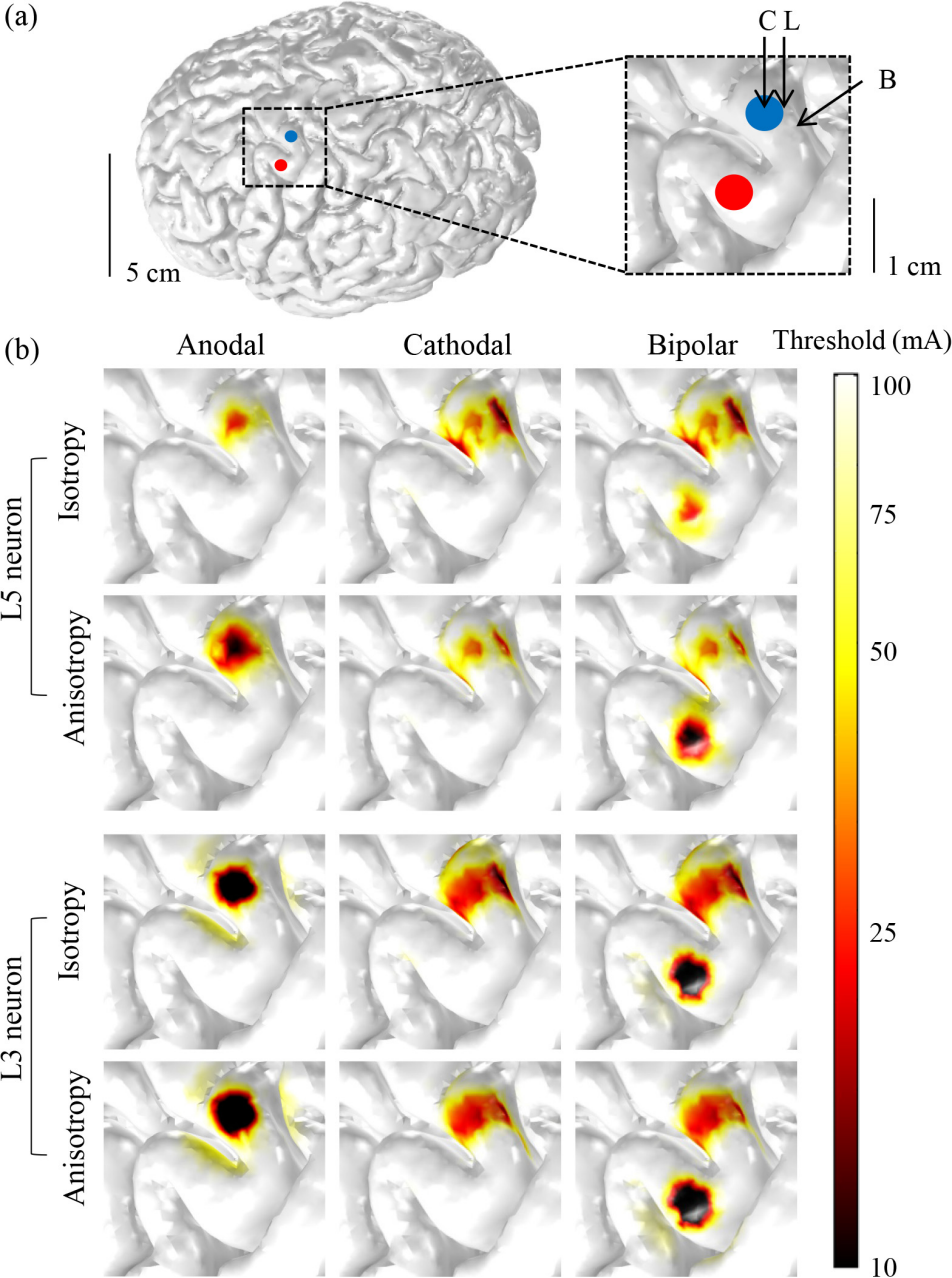
Comparison between isotropic and anisotropic conductivity for anodal, cathodal and bipolar stimulation. (a) Electrode placement in the anatomically realistic head model. Inset represents the ROI, including the crown (C), lip (L) and bank (B) on the GM. (b) The spatial extent of the excitation thresholds for L3 and L5 neurons between the isotropic and anisotropic models over three polarities.

The inclusion of anisotropy in the model changed the spatial extent of the excitation thresholds, as illustrated in Fig 4. L5 neurons in the anisotropic model were excited in a wider area around the crown during anodal stimulation and in a narrower, but deeper, bank area during cathodal stimulation than those in the isotropic model. Like L5 neurons, L3 neurons in the anisotropic model were activated more widely during anodal stimulation than those in the isotropic model, but they were activated slightly more in the deeper bank during cathodal stimulation. The overall change induced by anisotropy was smaller in L3 than in L5 neurons. This can be accounted for as follows: most L3 neurons were located within the GM, where the anisotropic WM conductivity may have minimal effects on L3 neurons.

**Fig 5:**
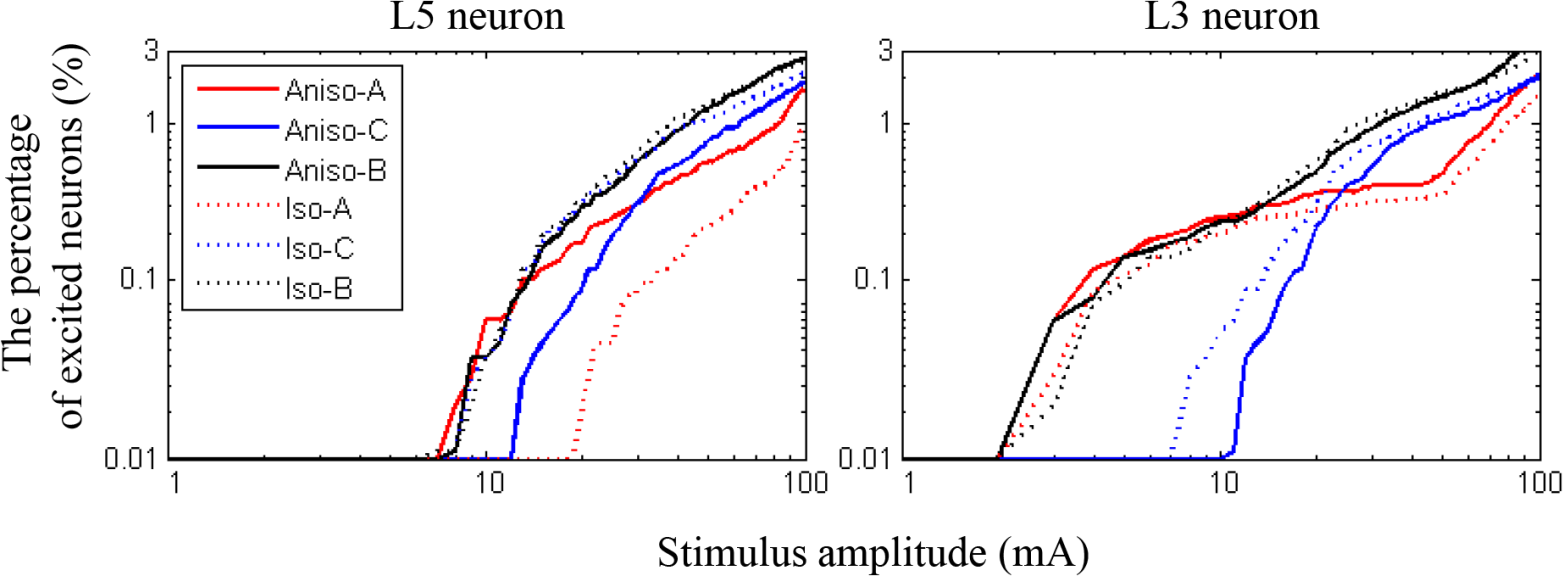
The relative ratio (%) of neurons excited with stimulation at three different polarities (anodal (A), cathodal (C), and bipolar (B) stimulation). L5 and L3 neurons in the anatomically realistic head model were compared between the isotropic (iso) and anisotropic (aniso) models.

Fig 5 presents the relative ratio of L5 and L3 neurons excited in the isotropic and anisotropic models as stimulus amplitude increased. In L5 neurons, the anisotropic model yielded substantially different results from the isotropic model: the anodal stimulation excited far more neurons, while the cathodal stimulation excited noticeably fewer neurons in the anisotropic model. L3 neurons showed behavior similar to those in L5, but the difference was quite small. This demonstrated the minimal effect of anisotropy on L3 neurons, but its relatively larger effect on L5 neurons. Interestingly, we observed that bipolar stimulation was not noticeably different between the anisotropic and isotropic models.

Table 3 illustrates the minimum excitation threshold (minimum input current to modulate neurons) for three different polarities in two models (the isotropic and anisotropic models). Similar to the results in Fig 5, the minimum thresholds of L5 neurons were quite different between models. The anisotropic model yielded a much lower minimum threshold with anodal stimulation and a higher threshold with cathodal stimulation than the isotropic model. However, the bipolar stimulation yielded a lower threshold between the two polarities (cathodal and anodal); As for L3 neurons, there was a small difference only with cathodal stimulation; the anisotropic model had a slightly higher threshold than the isotropic model.

**Table 3:** The minimum excitation thresholds (mA) over three polarities between the anisotropic and isotropic models; parentheses indicate the location of the neuron(s) excited.

polarity	L5 neuron		L3 neuron	
	Isotropy	Anisotropy	Isotropy	Anisotropy
Anodal	19 (C)	8 (C)	3 (C)	3 (C)
Cathodal	7 (B)	13 (B)	8 (B)	11 (B)
Bipolar	7 (B)	8 (C)	3 (C)	3 (C)

### Comparison among various anisotropic conductivities

Previously, we observed that L3 neurons are considerably less sensitive to WM anisotropy than L5 neurons. In this section, we investigated further the effect of anisotropic conductivities on L5 neurons. There are various principles that apply to the adaptation of anisotropic conductivity. In addition to the fixed value anisotropy, two principles of anisotropic adaptation were introduced: volume normalization anisotropy and volume constraint anisotropy with varying anisotropic factors (r = 2, 5, 10, and 100). A detailed description was presented in the section ‘Conductivity assignment.’

**Fig 6:**
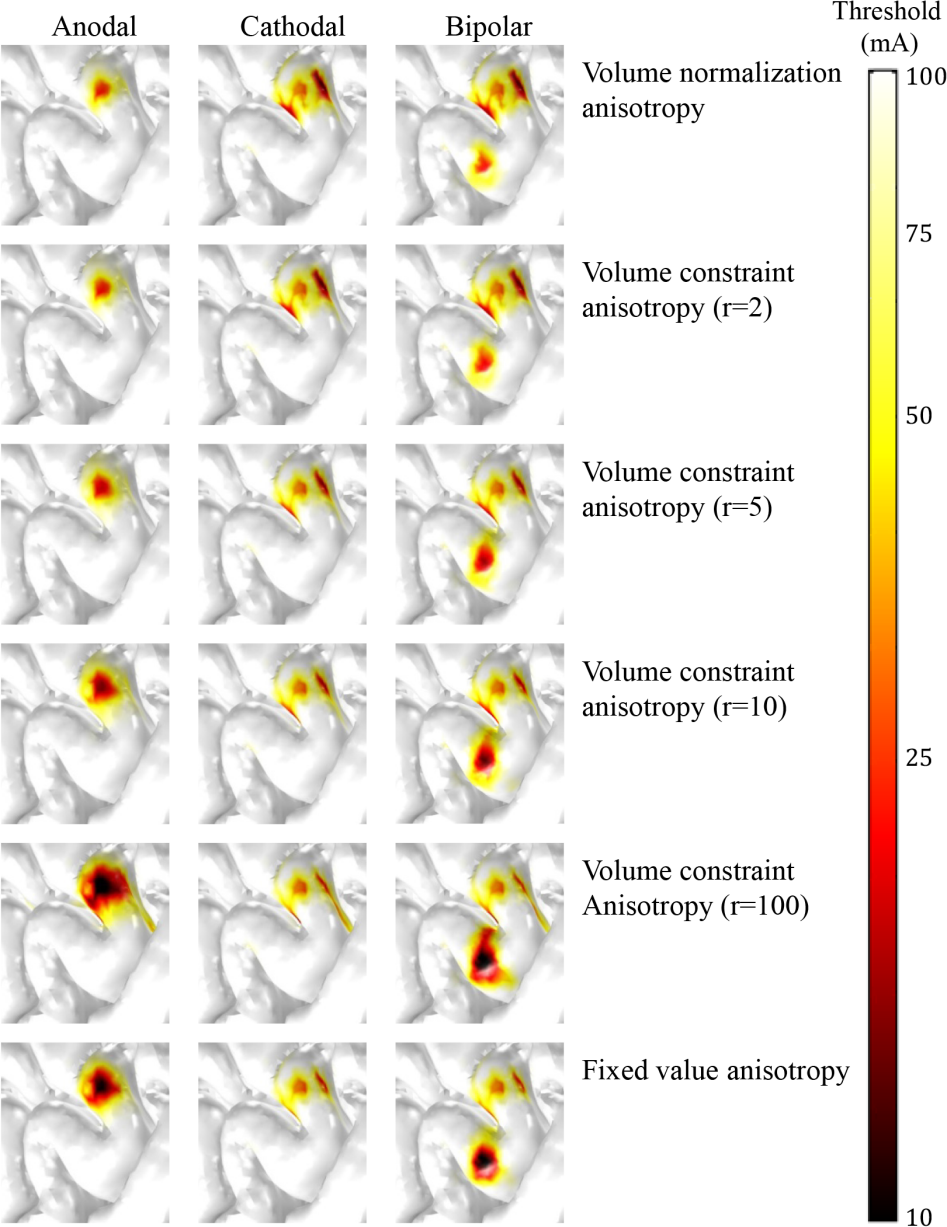
Comparison among various anisotropic conductivities for anodal, cathodal and bipolar stimulation. Spatial extents of excitation thresholds of L5 neurons are shown in various anisotropic models within the ROI shown in Fig 4(A).

Fig 6 illustrates the spatial extent of the excitation thresholds of L5 neurons over three polarities for the six anisotropic models. Volume normalization anisotropy and volume constraint anisotropy with r = 2 yielded almost identical behavior. As the anisotropic factor (r) in the volume constraint anisotropy increased, the anodal stimulation yielded a wider area of excitation. While the difference was quite small, the cathodal stimulation produced a slightly narrower area and seemed to activate neurons in the deeper bank as the anisotropic factor increased. Bipolar stimulation may yield the simple superimposition of anodal and cathodal stimulations.

**Fig 7:**
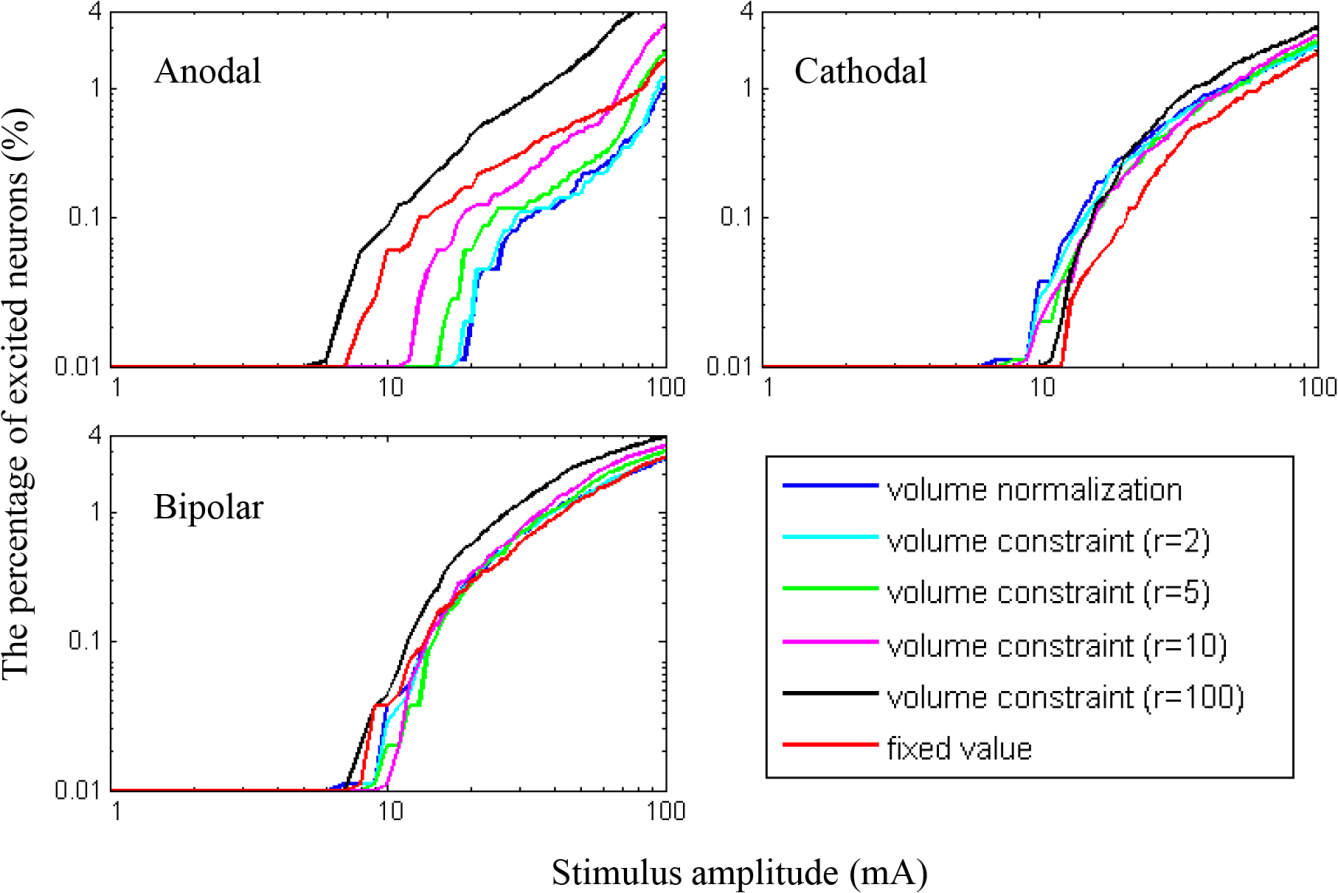
The relative ratio of the number of L5 neurons excited for three polarities. The six different anisotropic models were compared.

Fig 7 presents the relative ratio of L5 neurons excited over the six anisotropic models (volume normalization anisotropy, volume constraint anisotropies (r = 2, 5, 10, 100), and fixed value anisotropy). The overall behaviors of the relative ratio of L5 neurons excited were quite similar to the spatial extent of the thresholds (Fig 6). During anodal stimulation, both volume normalization anisotropy and volume constraint anisotropy with r = 2 yielded comparable ratios of neurons excited, and the number of neurons excited increased as the anisotropic factor increased. In cathodal stimulation, the numbers of neurons excited showed a very small difference over the anisotropic models, but conversely, they became slightly higher as the anisotropic factor decreased. When the impractical factor (r = 100) of volume constraint anisotropy was excluded, we found that the fixed value anisotropy yielded the greatest number of neurons excited in anodal stimulation and the fewest number in cathodal stimulation.

The minimum excitation thresholds of L5 neurons were estimated over the six anisotropic models, as tabulated in Table 4. It is clear that the higher anisotropic factor (r) of volume constraint anisotropy decreased the minimum threshold during anodal stimulation, while it increased the minimum threshold slightly during cathodal stimulation. Therefore, the minimum thresholds for the anodal stimulation decreased more than those for cathodal stimulation when r = 100. The crown was the primary area activated with the minimum threshold during anodal stimulation, while the bank was activated during cathodal stimulation. It is quite interesting that for high anisotropic factors (r = 5, 10, 100), the bipolar stimulation yielded slightly higher than minimum thresholds between the anodal and cathodal stimulations.

**Table 4:** The minimum excitation thresholds (mA) of L5 neurons in the anatomically realistic head model over the six anisotropic models; parentheses indicate the location of neuron(s) excited: crown (C) and bank (B).

Polarity	Norm	Vol (r = 2)	Vol (r = 5)	Vol (r = 10)	Vol(r = 100)	Fixed value
Anodal	18 (C)	18 (C)	16 (C)	12 (C)	6 (C)	8 (C)
Cathodal	7 (B)	8 (B)	8 (B)	9 (B)	11 (B)	13 (B)
Bipolar	7 (B)	8 (B)	9 (B)	10 (B)	8 (C)	8 (C)

## Discussion

### Effects of anisotropic conductivity on neuronal activation

To investigate the effects of the anisotropic model compared to the isotropic model, we introduced the principle of fixed value anisotropy, choosing eigenvalues reported by Wongsarnpiggon et al. [34,48]. During anodal stimulation, the anisotropic model activated neurons in a relatively wider area than the isotropic model; however, during cathodal stimulation, we observed conversely that a narrower area was activated in the anisotropic model than in the isotropic model. Consistently, the anisotropic model had the lowest minimum thresholds during anodal stimulation, while the isotropic model showed the lowest minimum thresholds during cathodal stimulation. These results produced by the anisotropic model matched well with previous studies [3,34,47,55–58], which reported that anodal stimulation had a lower minimum threshold than cathodal stimulation, while isotropic stimulation did not. We observed that anisotropic conductivity played a critical role in neuronal activation; such observations have been reported in studies on source localization or noninvasive stimulations [21,50,51].

To investigate further the effects of the anisotropic models on neuronal activation, we adapted two types of approaches to determine the anisotropic information derived from the DT-MRI. First, we looked at the volume normalization anisotropy, which normalizes conductivity tensors to compensate for variations in isotropic conductivity. Second, we investigated volume constraint anisotropy, which is used with a wide range of anisotropy ratios. We found that variation in the anisotropic information induced an altered spatial extent of the threshold and percentage of excited neurons, especially in anodal stimulation; however, it is still ambiguous which approach is appropriate. According to previous work [48,59], the anisotropic ratio of WM is known to be 1:10. However, due to the paucity of direct measurements of anisotropic conductivity [48] and the variation (both regionally and pathologically [21,60]) in the anisotropy ratios, the optimal method of taking into account WM anisotropy remains unknown to date.

### Comparison to existing modeling studies

Several previous modeling studies have investigated the effects of invasive cortical stimulation at the neuronal level [34,47,55,54] by developing a simplified extruded slab model and examining the influence of various parameters on neuronal activation. In the early stages, a small number of neuronal models were constructed on the precentral gyrus [47,55], and the leverage of model geometry, electrode placement and stimulus polarities were tested. More recently, a greater number of neuronal models and more varied types of neurons have been introduced in a larger cortical area [34,54]. Most of these studies have focused on the investigation of activated neural elements produced by ECS [34,47,55]. Furthermore, Zwartjes et al. [54] proposed stimulation protocols that target selected neuronal populations. Although they provided insight with respect to neuronal activation, information related to SuCS is difficult to predict due to the different placement of electrodes. Moreover, those neuronal models were coupled with the simplified extruded slab model, which represents the precentral gyrus only. Thus, in this study, we used the anatomically realistic head model with anisotropic conductivity derived from MR-DTI to yield more accurate extrapolation of neuronal activation, which may be distinct from earlier work.

Electrode location (on/under the dura mater), detailed neuronal morphologies, and computationally realistic brain models were also introduced distinctively in this work, but we observed results consistent with existing studies. During anodal stimulation, the crown, which lies beneath the active electrode, was the most excitable area, as most neurons in the crown were perpendicular to the electrode surface. In addition to the crown, cathodal stimulation favored excitation of the upper bank, where neurons are aligned primarily parallel to the electrode surface. Further, we found that axons were more excitable than soma and dendrites. These results are consistent with those reported in earlier studies [34,47,55,54].

It is interesting to note that the spatial extent of the excitation thresholds in bipolar stimulation was almost identical to the superimposition of both anodal and cathodal stimulations. This may be due to the large electrode center-to-center distance (13 mm in this work). It has been reported [34,47,55,54,61] that the anodal and cathodal fields during bipolar stimulation scarcely interfered with electrodes at least 10 mm apart, which is relevant to our results.

### Comparison with empirical data

Our empirical results showed consistently that anodal stimulation activated pyramidal neurons directly at lower amplitudes than cathodal stimulation [3,56–58]. Further, Hern et al. reported that in corticofugal fibers, cathodal stimulation elicited excitation thresholds 1.5–5 times higher than anodal stimulation [57]. Comparing the minimum excitation thresholds in the anatomically realistic head model with fixed value anisotropy, anodal stimulation was 8 and 3 mA for the L5 and L3 neuronal models, respectively, while cathodal stimulation was 13 and 11 mA. Thus, cathodal stimulation was 1.6 and 3.7 times higher than anodal stimulation for L5 and L3 neurons, respectively. These results reconfirm the fact that anodal stimulation produces lower current thresholds than cathodal stimulation, and these ratios agree well with the results of other experiments [52]. Further, we found that pyramidal neurons in the lip of the central sulcus responded at lower thresholds in cathodal than anodal stimulation, which is consistent with the results of Phillips’ experiment [56].

Responses in the pyramidal tract following cortical stimulation are known to be classified into D- and I- wave responses [56,58,62]. The initial positive deflection is interpreted as a D-wave, which is likely produced by direct activation of cells in the motor cortex. Next, a series of variable positive deflections of the I-wave follow from synaptic excitation and/or re-excitation of pyramidal cells with longer latencies. According to Gorman [56], at the threshold of stimulating intensities to evoke neuronal responses, the D-wave was elicited in anodal stimulation, while cathodal stimulation evoked I-waves. At suprathreshold stimulating intensities, both anodal and cathodal stimulation produced D- and I-waves. At supramaximal stimulation, cathodal stimulation produced larger amplitude D- and I-waves than anodal stimulation. In this work, we explored the individual neuronal responses to determine the cellular target of stimulation, so that we could observe direct responses (D-wave) only. The rate of neurons excited in the anisotropic model well represented the comparison between polarities, as shown in Fig 5. When anodal stimulation began to trigger action potentials, cathodal stimulation did not. Moreover, anodal stimulation excited more neurons at lower amplitudes than cathodal stimulation. However, by increasing the stimulus amplitude, cathodal stimulation evoked action potentials in more neurons than anodal stimulation. These results are consistent with previous experimental observations of the D-wave [51].

Wongsarnpigoon et al. studied the effects of electrode position and geometry on neuronal activation by ECS [34]. Before beginning the study, they validated a compartmental pyramidal neuronal model located in the crown by comparing it with experimental data. In this work, we employed the pyramidal neuronal models used in Wongsarnpigoon et al. [34], which were modified versions of cat visual cortex [52] fitted to human brain geometry. These provided a valid rationale of the simulation results that well matched those in our study.

### Limitations and future steps

In our model, we considered the L5 and L3 pyramidal neuronal models; their morphologies and electrical properties were taken from cat visual cortex [52], because to our knowledge the properties and morphologies of most human cortical neurons have not been described well thus far. Due to this uncertainty with regard to neuronal properties, computational results may lead to inaccurate neuronal responses. For example, in the previous modeling study, the presence of collaterals reduced the excitation threshold by 50% [63], and even evoked action potentials in neurons that were perpendicular to the electrode during cathodal stimulation [54], while unbranched neurons were not activated [61]. Despite this possible discrepancy in the neuronal models, we reproduced quite reasonable computational results that are comparable to experimental data. As a consequence, we expect that excitation thresholds induced by SuCS may be estimated reasonably through our computational model.

We constructed individualized and non-communicating populations of neurons. This limits the observations of the I-wave that is followed by indirect and trans-synaptic activation. The reason for investigating the I-wave is that, under certain conditions, cathodal stimulation activated cellular targets more readily than anodal stimulation [2]. This implies that motor cortex stimulation activates cortical neurons largely indirectly. Although observing multiple interconnected neurons is important in indirect responses, the response of a single pyramidal neuron is also useful [58]. Because these neurons are activated directly from the common path issuing from the cortex, they may give evidence of which neurons are recruited by the stimulus-induced electric field.

There were several limitations in our modeling study with the anatomically realistic head model: first is the substrate shape inserted. In this work, we only considered substrates surrounding electrodes. In order to investigate whether the substrate shape affects neuronal excitation, we constructed two types of electrodes in the simplified extruded slab model. One was a 5 × 18 mm^2^ sized strip-type electrode and the other was a disc-type electrode with covered substrates that were designed for this study. Then, we compared the excitation thresholds of L5 neurons during bipolar stimulation, and the thresholds differed only up to an average of 0.37%, which is negligibly small. Even though the complex brain geometry of the anatomically realistic head model may be affected by these changes, it is also possible that it has only a minor influence. Another modeling issue is the construction of the dura mater. Due to the extreme thinness of the dura mater, we were not able to include it in the anatomically realistic head model. However, because electrodes in SuCS are located under the dura mater and we focused on activation within the cortex, the exclusion of the dura mater may be permissible. We underpinned it by comparing the extruded slab model with and without dura mater, which showed 0.003% differences in the excitation thresholds of L5 neurons during bipolar stimulation. Dura mater construction is necessary to consider ECS further, as it is used more widely than SuCS. Thus, the inclusion of the dura mater in both ECS and SuCS computational studies will be considered in future work.

## Conclusions

We assessed the effects of an anatomically realistic head model with isotropic/anisotropic conductivities on the activation of pyramidal neurons. With anisotropic conductivity, there was a strong influence on L5 neurons compared to L3 neurons, as their axons stretched to the WM. The crown was the most excitable area, and during cathodal stimulation, the bank showed greater activation at the lowest excitation threshold. The anatomically realistic model with fixed value anisotropy yielded results quite consistent with empirical data, such that anodal stimulation activated neurons at a lower excitation threshold than cathodal stimulation; however, results from the isotropic model were not consistent. The spatial extent and excitation thresholds varied according to principles of anisotropic conductivity adaptation. In conclusion, anisotropic conductivity has a strong influence on simulating neuronal activation and, by comparison to the isotropic model, it may improve the prediction of the stimulation effects produced by SuCS.
