# Correction: Computational Study of Subdural Cortical Stimulation: Effects of Simulating Anisotropic Conductivity on Activation of Cortical Neurons

**DOI:** 10.1371/journal.pone.0134422

**Published:** 2015-07-28

**Authors:** The PLOS ONE Staff

There are errors in the Funding section. The correct funding information is as follows: This work was supported by the National Research Foundation (NRF) funded by the Korean Government (NRF-2010-0026438), PLSI supercomputing resources of Korea Institute of Science and Technology Information (KISTI), and a grant from the Integrative Aging Research Center of Gwangju Institute of Science and Technology (GIST). The publisher apologizes for the errors.
